# Adolescent girls in aquaculture ecozones at risk of nutrient deficiency in Bangladesh development and validation of an integrated metric

**DOI:** 10.1186/s12889-023-15175-z

**Published:** 2023-02-28

**Authors:** Eleanor Grieve, Abdullah-Al Mamun, Baukje de Roos, Benoy K. Barman, Gulshan Ara, Nanna Roos, Alexandra Pounds, Alan A. Sneddon, Francis Murray, Tahmeed Ahmed, David C. Little

**Affiliations:** 1 1 Lilybank Gardens, Institute of Health and Wellbeing, University of Glasgow, Glasgow, G12 8RZ UK; 2 Department of Fisheries and Marine Science, Noakhali Science and Technology University, University Road, Noakhali, 3814 Bangladesh; 3 The Rowett Institute, University of Aberdeen, Ashgrove Road W, Aberdeen, AB25 2ZD UK; 4 WorldFish, Bangladesh and South Asia, House 355/A Rd 114, Dhaka, 1212 Bangladesh; 5 Nutrition and Clinical Services Division, International Centre for Diarrhoeal Disease Research, GPO Box 128, Dhaka, 1000 Bangladesh; 6 Department of Nutrition, Exercise and Sports, University of Copenhagen, Nørre Allé 51, 2200 Copenhagen, Denmark; 7 Institute of Aquaculture, University of Stirling, Stirling, FK9 4LA UK

**Keywords:** Adolescent girls, Nutritional status, Anthropometry, Agrosystems, Aquaculture, Sustainable livelihoods, Nutrient deficiency, Omega-3 index, Female autonomy

## Abstract

**Background:**

This study developed and validated an integrated metric that enhances understanding of linkages between agro-ecological and socio-economic factors that are important for explaining nutritional wellbeing in relation to fish consumption; especially among adolescent girls who are at risk of nutritional deficiency in Bangladesh. Currently, there is no metric that takes account of environmental, cultural and economic contexts when considering fish consumption and dietary health from a policy perspective.

**Methods:**

The study was designed as a bi-seasonal survey, repeated in the same population of adolescent girls recruited during the dry and wet seasons. Sampling was stratified by five settings (four aqua-agroecological zones and one processing plant community), with 60 girls recruited in each. Associations between candidate predictors (salinity, diet diversity, religion, socio-economic status and women’s autonomy score) and dependent variables representing nutritional outcomes (anthropometry, omega-3 index and micronutrient levels) were explored in multivariable regressions. The fitted model with its predictors was validated, and a risk score derived from responses to a few short questions on religion, salinity zone, female autonomy, diet diversity and tilapia consumption.

**Results:**

The omega-3 index showed the clearest distinction between seasons, by salinity and religion. Higher female autonomy, religion (being Hindu rather than Muslim), geographical location (living in a high or mid-saline area), and a higher dietary diversity were the strongest predictors of whole blood omega-3 index. The c-index for the prognostic model was 0.83 and 0.76 in the wet and dry season respectively, indicating good predictive accuracy. There appeared to be a clear trend in risk scores differentiating between those ‘chronically at risk’ and those ‘never at risk’.

**Conclusions:**

Observational data on different aquaculture-ecozones defined by salinity enabled us to establish linkages between seasonal fish intake, religion, diet diversity, female autonomy and nutritional wellbeing. The purpose of the metric is to reveal these specific linkages in practice. This tool should improve targeting of timely, preventative and cost-effective nutritional interventions to adolescent girls most at-risk from low omega-3 levels in communities where seafood is produced.

**Supplementary Information:**

The online version contains supplementary material available at 10.1186/s12889-023-15175-z.

## Background

Aquaculture is a fast-growing food production sector in low-income and food-deficit countries with aquatic ecozones [1, 2]. Yet, its specific impact on the nexus of nutritional status, food security and livelihood in local communities where commercial, and particularly export-orientated aquaculture activities are developed, is largely unknown [1]. Evidence that commercial aquaculture in Low- and Medium-Income Countries (LMIC) can have important effects on local livelihoods has not been matched with detailed studies of its direct impacts on peoples’ nutritional status, health and well-being [1, 3]. In Bangladesh, fish is an important protein component of the diet but household level intake data typically overestimates actual seafood consumption and does not account for varying intakes based on sex, age, income and religious beliefs [4]. Adolescent girls represent a vulnerable group in Bangladesh, with higher nutritional needs relative to energy requirements than other adult household members, and at the same time likely to have restricted access to food. For this group, an optimal diet is critical for their own health and – in the case of early marriage and motherhood – for the *in-utero* growth of the foetus and for breast-feeding (the critical ‘1000 days’) [1, 5]. Greater women’s autonomy, which has been found to confer improved food and resource allocation within the household [6], and has been strongly linked to women’s employment, especially outside the home [1, 7], may be linked to differentiated health outcomes by sex [8]. Differences in autonomy may explain why women’s participation in the labour force differ between Muslim and non-Muslim communities [9]. Given the significant increase in women in employment related to export-led processing of farmed seafood, we need a seafood value chain approach to characterise the developmental dimensions and interdisciplinary nature of improving adolescent welfare linking information on aquaculture production systems, food availability, dietary intakes, nutritional status and individual health [1, 2]. Yet, policies addressing the specific challenges of risk management of these communities is limited by the sectoral separation of aquatic food production the fisheries and aquaculture sector—and health, meaning there is a disconnect between professionals on all levels responsible for fisheries and aquaculture, and those tasked to support public health and nutrition initiatives [2].

The agro-ecological dynamics in coastal-estuarine zones and seasonality are also determining factors for health and wellbeing outcomes [10, 11]. In Bangladesh, the aquatic environment ranges from coastal fully saline to inland freshwater zones. Seasonal and annual fluctuations in freshwater supply create a variable salinity gradient from coast to inland that impacts on aquatic food production and on food production more generally [1]. A clear relationship was found between the aquatic environments defined by coastal to inland salinity zones and whole blood omega-3 levels, a marker of fish intake. The mean omega-3 index was > 4.0% in high saline areas and < 2.8% in freshwater areas, assumed to reflect the fatty acid composition of the fish consumed combined with the amounts of fish consumed. In addition, the omega-3 index was higher during the wet season compared to the dry season, similarly indicating dietary diversity and access to omega-3 rich fish were higher during the wet season. Therefore, the omega-3 index appeared to be a sensitive biological indicator for variation in the specific food environment in Bangladesh [10, 11]. Other nutritional markers such as mid-upper arm circumference (MUAC) or body-mass index (BMI), did not correlate with the aquatic food environment [10, 11].

This study aimed to develop and validate an integrated metric that i) enhances our understanding of linkages between agro-ecological and socio-economic factors that are important for explaining the role of fish intake in nutritional wellbeing and ii) supports more targeted and sensitive identification of nutritional wellbeing of those at risk of nutritional deficiency such as adolescent girls in Bangladesh.

## Methods

The model is reported in line with the Transparent Reporting of a multivariable prediction model for Individual Prognosis or Diagnosis (TRIPOD) statement [12].

### Study setting

The study setting was the dynamic shrimp growing environments of the Greater Khulna region of southwest Bangladesh where aqua-agricultural systems are export-orientated, based mainly on farmed shrimp and prawn production. Households were identified in community clusters involved in farmed seafood value chains, based on agro-ecologies that characterise the prevailing salinity gradient of the region; high, medium and low-saline, freshwater and an urban location with a concentration of shrimp processing facilities that were identified previously [10, 11].

### Study design and sampling

We designed the study as a bi-seasonal survey conducted in the same 300 adolescent girls aged 12 – 16 years old recruited during the dry season (August–September 2017) and wet season (April–May 2018) to capture seasonal variations in fish availability. The wet season is associated with peak shrimp production, and the dry season with peak fish and prawn production. A multistage random sampling procedure was followed to ensure a random selection of girls from high saline (HS), medium saline (MS), low saline (LS) and fresh water (FW) agro-ecological areas, and within these areas, unions (small administrative units) and wards. Sampling was stratified by the sefour aqua-agroecological zones and one processing plant (PP) community, with 60 adolescent girls recruited in each site. Each of the wards was divided into a number of 400-household segments from which the adolescent girls were recruited, with recruitment being stratified aiming for an equal number of thirty households identifying themselves as Hindu or Muslim, at each site. However, in the PP community, all included households were identified as Muslim. Although at the national level, Hindu communities represent less than 10% of the population, the proportion of Hindus in the study areas was high. Households with at least one unmarried adolescent girl were identified and invited to participate in this study.

### Data collection

Data were collected by a trained team of female enumerators with a background in food and nutrition from Khulna Collegiate Women’s College, an affiliate institute of Khulna University, and recruited by Noakhali Science and Technology University (NSTU). Two health technologists were also recruited for the collection of biomarker samples. The field supervisors and data collection teams were trained on the interview techniques, questionnaires, and nutritional status measurements. A pre-tested survey tool (see Supplementary Material) was developed for use at the household and individual level to assess socio-economic status, dietary intake and women’s autonomy. Nutritional status was assessed by anthropometric and biochemical (blood and urine) markers. Repeated data collection on socio-demographic information and anthropometry in a random selection of 5% of the study participants was undertaken, and identical forms, equipment, definitions, and methods were used throughout. Details of the data collected are described below.

### Demographics and socio-economic status

Indicators included information on ethnicity, religion, level of education and occupation of household head, number of household members, and ownership of the house.

### Dietary intake

A 24-h dietary recall method was used to measure the dietary diversity of adolescent girls and a 7-day semi quantitative Food Frequency Questionnaire (FFQ), which had been previously developed and validated [10, 11], was used to assess types of seafood and food rich in omega-3 PUFA and iron [12]. Photographs of the serving and amount (grams) were used to assess the quantity consumed. Raw food weight was calculated by using appropriate conversion factors [13]. All food items were grouped into ten major food groups based on standard approaches [14–16]: cereals (boiled rice, puffed rice), vegetables (plants, vegetables, leafy vegetables), pulses (pulses and legumes), meat (chicken and meat products), milk (milk and milk products), beverage (tea, coffee), eggs, fruits and others (ice-cream, chocolate). At the household level, fish intake (seafood, finfish and shellfish) was also ranked by type of fish most commonly consumed based on participant recall for the past 5 days, 3 months and 1 year. Micronutrient status was solely assessed by measuring levels of serum 25-hydroxyvitamin D_3_, ferritin and retinol, and urinary iodine (see Biomarkers below).

### Female autonomy

A participatory method to measure women’s autonomy was adapted to the population-specific setting through focus groups prior to the survey [17]. Twelve focus group discussions, each consisting of 10 -12 girls,, were facilitated. Participants were adolescent girls (12–16 years) in seafood farming communities in the saline floodplain area of southwest Bangladesh. A previously developed index using information on household assets was adapted as a measure of socio-economic status [10, 11]. Individuals were selected to be representatives of ‘better-off’ (rich and medium categories) and ‘worse-off’ (poor and ultra-poor) households from Muslim and Hindus communities. Major domains (influencing factors) of women’s autonomy including access to nutritional knowledge and food, mobility and social security, mental health and ambition were considered during the focus group discussions. A total of 32 questions covering 7 domains were developed for inclusion in the survey (see Supplementary Material).

### Anthropometric indices

Anthropometric data on height (cm) and weight (kg) were collected. Anthropometric instruments were pre-tested among a volunteer population. The girls were weighed using electronic scales (Tanita Inc. Tokyo, Japan) with a precision of 100 g. Height was measured using locally made standardised wooden length/height boards with a precision of 0.1 cm. As stated above, field supervisors independently repeated the data collection on anthropometry in a random selection of 5% of the study participants. Mid-upper arm circumference (MUAC) tape was used to determine MUAC (mm) [18]. The mean of three consecutive measurements was considered as the observed value and recorded.

### Biomarkers

Biological samples, including blood and urine, were collected to assess serum levels of 25-hydroxyvitamin D_3_, ferritin, retinol, the inflammatory markers C-reactive protein (CRP) and alpha-1-acid glycoprotein (AGP), and urinary iodine concentration. Analysis was performed in the Nutritional Biochemistry Lab at icddrb, Bangladesh. Fatty acid composition in whole blood samples taken from a finger prick was measured and analysed using established methods [19] in dried blood spots. The omega-3 index was expressed as total eicosapentaenoic acid (EPA) + docosahexaenoic acid (DHA) as a ratio of total fatty acids in whole blood.. Total 25-hydroxyvitamin D level was measured by electrochemiluminescence binding assay using a Roche Cobas e601 automated immune analyzer [20]. The assay employs a polyclonal antibody directed against 25-hydroxyvitamin D [21]. Urinary iodine was determined by a colorimetric method at the icddr,b [22]. Serum ferritin, CRP and AGP were analyzed by a sandwich ELISA technique [23]. For further details, see Supplementary Material.

### Selection of candidate variables for the model

#### Outcome variables

Anthropometry measures, micronutrient status and the omega-3 index were considered as measures of nutritional wellbeing and incorporated into a multivariate multiple regression model. A multivariate regression model was fitted in order to allow for several dependent variables (anthropometry, omega-3 index and micronutrient levels) with the same independent variables. This requires these outcome variables to be correlated to some degree and have a normal distribution. We used standard anthropometric measures for classifying nutritional status. For MUAC, both 210 mm and 185 mm were used, the latter being a cut-off which may be considered relevant for Asian adolescent girls^1^[24]. BMI was classified from ‘obese’ (> 30 BMI kg/m^2^) to ‘grade III thinness’ (< 16 BMI kg/m^2^) [25]; We also used age-adjusted z-scores for BMI^2^ [26] and Asian population BMI cut-offs in a sensitivity analysis [27]. Based on cardiovascular risk, an optimal target level of the omega-3 index is ≥ 8%, and an undesirable level is less than ≤ 4%, with 4–8% being an intermediate risk zone for cardiovascular disease [28]. This was treated as both a continuous and categorical variable, classified into high and low cardiovascular risk using a threshold set to < 3%, adjusting for the fact the fatty acid composition was measured in whole bloods [29]. As stated above, micronutrient status was solely assessed by measuring serum 25-hydroxyvitamin D_3_, ferritin and retinol, and urinary iodine levels. We did not measure intake of these micronutrients but were measuring levels in serum/urine. A micronutrient score was based on a count of how many of these four micronutrients were above or below a threshold indicating an adequate level (0 for below and 1 for above). A girl could therefore have a score of 0 to 4. Adequate threshold values for serum 25-hydroxyvitamin D_3_, ferritin and retinol, and urinary iodine levels were set as follows: 25-hydroxyvitamin deficiency at the population level was defined as a median serum level < 50 nmol/L [30]; Iron deficiency (ID) at the population level was defined as a median serum level < 15 mmol/L, after adjusting for inflammatory markers CRP and AGP [31]; the threshold for vitamin A deficiency (VAD) was a serum retinol concentration of < 0.7 µmol/L and severe VAD at < 0.35 µmol/L; Urinary iodine deficiency (UID) was defined as an excretion level (< 99 µg/L)^3^ [32].

### Candidate predictors of wellbeing

Nutritional wellbeing predictors are those considered to have potential to influence the nutritional wellbeing outcomes as described above. These were informed by findings on associations of key livelihood impacts with different salinity gradients [10, 11]. Limited off-farm livelihood opportunities in medium salinity and freshwater areas pushed more low social economic households into aquaculture, with net returns from aquaculture significantly lower in these communities [10, 11]. A greater dependence on aquaculture was mainly explained by limited off-farm livelihood options related to their greater geographical isolation and travelling times to mangroves and urban areas compared to high and low salinity communities, respectively. However, in low salinity and freshwater communities, dyke vegetable and rice production were important parts of farmer livelihoods. Household and individual dietary diversity scores were calculated as a proxy measure of household food access in the past 24 h based on the 10 food groups listed above using a simple score on amount consumed. A female autonomy score was created as a key indicator of resource allocation within the household. Responses were summed to produce a total score by each domain. The scores of each of the domains were then aggregated, assigning equal weights to each domain [17, 33]. Socio-economic status was split into wealth quintiles based on an asset score from poorest to richest.

### Statistical analysis

The sample size was based on standard sample size calculations to power for 80% (Zα: 1.96, design effect: 1.2) resulting in a sample size of 295, rounded to 300 adolescent girls to allow for 60 adolescent girls to be selected from each of the 5 sites. Data collected for the same adolescent girls in the dry and wet season were incorporated into the multivariate multiple regression model.

Model performance was undertaken using measures of calibration and discrimination. Calibration was investigated by plotting the observed against the predicted proportions of girls ‘with’ the outcome. Groups of girls with similar predicted probabilities were constructed by splitting them into deciles. The actual proportion with the outcome in each group was then plotted against the group (average) probability. This calibration plot was accompanied by the Hosmer–Lemeshow ‘goodness of fit’ test. Discrimination, namely the sensitivity and specificity of the model, was carried out using the area under the Receiver Operating Characteristic (ROC) and the c-statistic (concordance) index. Various statistics can summarise discrimination between individuals with and without the outcome, in this case those below the threshold of < 3% for omega-3 index levels. The ROC or the c-statistic index is the chance that given two girls, one who will be identified as having an omega-3 index < 3% and the other who will not, the model will assign a higher probability of this status to the former. It plots the sensitivity (true-positive rate) against 1 – specificity (false-positive rate) for consecutive cut-offs for the predicted risk [34]. The larger the area-under-the-curve (AUC), the better with 0.5 being as good as random assignment and 1 being perfect.

Finally, the fitted model with its predictors was validated. As the development dataset was too small to put aside a portion for internal validation, bootstrapping with replacement was applied. We also validated the model using external data. These data were collected from a different cohort of adolescent girls in the same geographic setting during the dry season (February to March 2019). For the validation data, we used a rule of thumb for logistic regression of 10 observations per number of ‘girls at risk’ (omega-3 index < 3%) [35]. Validation data were collected only for those variables identified for inclusion in the final metric, incorporating religion, salinity zone and questions on women’s autonomy, diet diversity, fish consumption and basic descriptors of the population. All analyses were undertaken using STATA statistical software version 14.

## Results

### Characteristics of the population

We collected data from 298 adolescent girls during the dry season and from 270 adolescent girls in the wet season. Characteristics of the population are shown in Table 1. The validation data were collected from 103 girls, with the same average age (14 years) and distribution across salinity zones and religion.

**Table 1:** Background characteristics of adolescent girls by salinity areas at the 1st survey (dry season)

Religion	Muslim	Hindu	Muslim	Hindu	Muslim	Hindu	Muslim	Hindu	Muslim
**Salinity area**	**High saline**		**Medium saline**		**Low saline**		**Fresh water**		**Processing Plant**
**dry**	**n = 28**	**n = 32**	**n = 31**	**n = 29**	**n = 28**	**n = 30**	**n = 30**	**n = 30**	**n = 60**
**(wet)**	**(n = 28)**	**(n = 28)**	**(n = 26)**	**(n = 28)**	**(n = 25)**	**(n = 28)**	**(n = 28)**	**(n = 29)**	**(n = 50)**
Age, years (mean ± sd)	14.2 ± 1.5	13.9 ± 1.3	14.0 ± 1.3	13.7 ± 1.5	14.3 ± 1.6	14.0 ± 1.4	14.2 ± 1.6	14.1 ± 1.5	13.3 ± 1.2
Schooling, years (mean ± sd)	7.0 ± 1.8	7.5 ± 1.7	7.12 ± 1.8	7.1 ± 1.8	7.5 ± 2.1	7.16 ± 1.9	7.5 ± 1.9	8.0 ± 1.8	6.2 ± 2.0
Household size, number (mean)	5.3	5.1	4.9	5.0	4.4	4.6	5.0	4.7	4.6
Wealth quintiles									
1^st^ quintile (poorest)	15 (27.3%)		15 (29.4%)		13 (24.1%)		3 (5.0%)		5 (15.6%)
2^nd^ quintile	6 (10.9%)		13 (25.5%)		10 (18.5%)		13 (21.7%)		8 (25.0%)
3^rd^ quintile	12 (21.8%)		5 (9.8%)		12 (22.2%)		19 (31.7%)		3 (9.4%)
4^th^ quintile	14 (25.5%)		10 (19.6%)		8 (14.8%)		10 (16.7%)		8 (25.0%)
5^th^ quintile (richest)	8 (14.6%)		8 (15.7%)		11 (20.4%)		15 (25.0%)		8 (25.0%)

### Model development

Unadjusted and adjusted associations using multivariate regressions between candidate predictors (salinity, diet diversity, religion (categorical by Hindu/Muslim), socio-economic status (categorical by wealth quintiles) and women’s autonomy score) and nutritional outcomes denoted by our identified endpoints (anthropometry, omega-3 index, and micronutrient levels) were explored. We also looked at health outcome correlations by season, and specifically between BMI and MUAC with mean omega-3 index level, and MUAC with micronutrient levels and composite score (iodine, ferritin, 25-hydroxyvitamin D_3,_ retinol) all by geographical location (salinity area). The omega-3 index showed the clearest distinction between seasons, by salinity and religion. Anthropometry and micronutrient status showed little correlation with the omega-3 index or associations with the pre-specified variables and these were not investigated further^4^ (see Supplementary Materials) The outcome of the model was, in turn, defined by the omega-3 index. After analyses, predictors that remained in the risk prediction model were simplified as follows: as the score for autonomy of adolescent girls was principally driven by girls’ knowledge of food and the freedom to participate in recreational activities, only those domains were included; little association was found with socio-economic status when included in a multivariable regression, so this was omitted; for fish intake, we used only tilapia consumption as this was one of the most commonly consumed fish. It is present across the saline transect and, with a high correlation of the consumption of this species to location, tilapia consumption was considered a proxy for dietary intake of omega-3 fatty acids [10, 11]. These binary, categorical and continuous variables, with a mix of stronger and weaker associations, were included in the final model.

### Model results

Keeping the omega-3 index as a continuous variable, the variability explained (R-squared) by the full linear regression model suggests that there is a difference in omega-3 index levels between salinity areas and religion, with the model’s inputs explaining more of the observed variation in the wet season (R^2^ = 0.46) than in the dry (R^2^ = 0.27). Although religion and autonomy were found to be aligned (and autonomy falls out of the full model from being significant when religion is included due to co-linearity), we included both in the model as combined they accounted for greater variation in the omega-3 index. Using omega-3 index cut-offs as the measure of outcome, 23% of girls in the wet season and 39% of girls in the dry season had an omega-3 index < 3% (adjusting for the fact these were measured in whole bloods) [29]. The logistic model showing all regression coefficients to allow predictions for each girl is shown in Table 2.

**Table 2:** Final prediction model

	Dry season				Wet season			
	Dependent variable:							
	Omega-3 index < 3%							
	Co-efficient (SE)	OR	95% CI		Co-efficient (SE)	OR	95% CI	
Religion (Hindu)	0.752 (0.32)**	**2.12**	1.13	4.00	1.823 (0.41)***	**6.18**	2.75	13.93
Diet Diversity score (girl)	0.256 (0.16)	1.29	0.94	1.77	-0.322 (0.16)**	**0.72**	0.53	0.98
Female Autonomy (mobility)	-0.103 (0.05)**	**0.90**	0.81	1.00	0.017 (0.09)	1.02	0.84	1.22
Female Autonomy (food choice)	-0.077 (0.14)	0.93	0.71	1.21	0.022 (0.19)	1.02	0.70	1.49
tilapia consumption	-0.021 (0.09)	0.98	0.81	1.18	0.333 (0.13)**	**1.40**	1.08	1.80
Salinity—medium	2.035 (0.56)***	**7.65**	2.54	23.06	2.35 (0.76)***	**10.50**	2.39	46.17
Salinity—low	0.220 (0.44)	1.25	0.53	2.93	-0.802 (0.51)	0.49	0.17	1.22
Freshwater	-0.885 (0.42)**	**0.41**	0.18	0.94	0.981 (0.55)*	**2.67**	0.91	7.85
Processing plant	-0.409 (0.43)	0.66	0.29	1.54	3.544 (0.86)***	**34.60**	6.40	186.98
Constant	0.169 (0.64)	1.18	0.34	4.18	0.586 (0.91)	1.80	0.30	10.76
Observations	295				260			

Notes:Statistically significant at *p < 0.1; **p < 0.05; ***p < 0.01

Log likelihood (dry) = -166.26, LR chi2 = 61.09, Prob > chi2 = 0.000

Log likelihood (wet) = -99.92, LR chi2 = 78.64, Prob > chi2 = 0.000

### Model performance

**Fig. 1:**
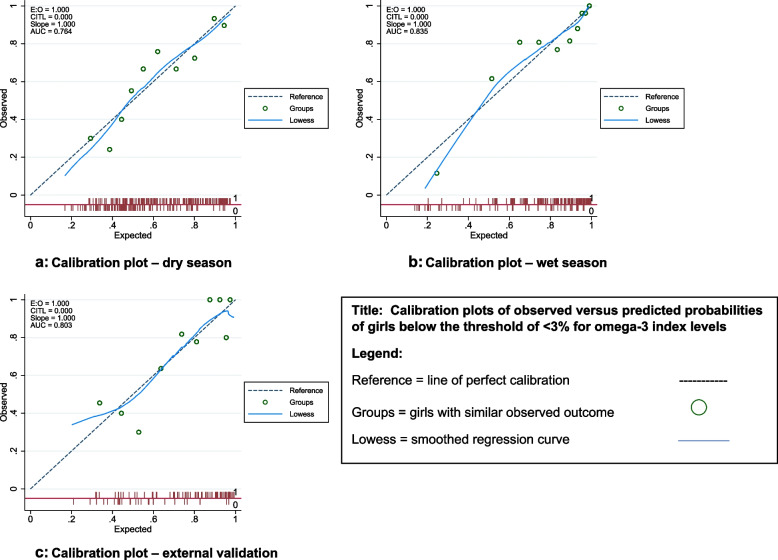
Calibration plots of observed versus predicted probabilities of girls below the threshold of < 3% for omega-3 index levels. Legend: Reference = Line of perfect calibration. Groups = Girls with similar observed outcome. Lowess = smoothed regression curve. Figure 1**a**: Calibration plot – dry season. Figure 1**b**: Calibration plot – wet season. Figure 1**c**: Calibration plot – external validation

Results showed Hosmer–Lemeshow goodness of fit test for dry and wet season of *p* = 0.24 and 0.21 respectively, indicating that the model fitted the data and that observed probabilities were not significantly different from predicted probabilities (see Supplementary Materials). The calibration plots are shown in Fig. 1 (a) and (b). The *c* index for a prognostic model is typically between 0.6 and 0.85. The AUC/c-index in the wet season was 0.83 and in the dry season 0.76, indicating good predictive accuracy. These results indicate the model performed better in the wet compared with the dry season.

### Model validation

Based on a third round of field data and bootstrapping the original data set, the metric was validated. In the validation dataset, 28% (*n* = 29) had omega-3 index levels below the risk threshold of < 3%. Results on the performance of the model using the external validation data were similar to the results obtained from the development data, with AUC/c-index equal to 0.80. The calibration plot is shown in Fig. 1(c).

### Metric risk scores

**Fig. 2:**
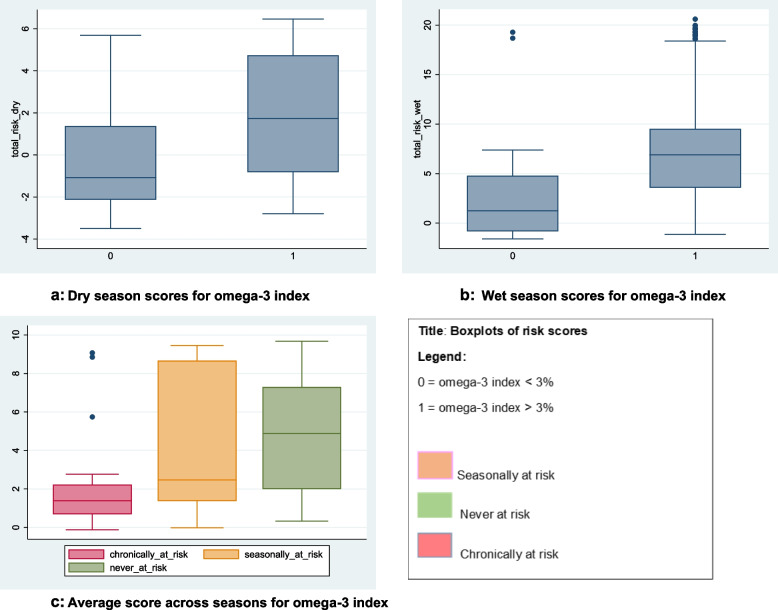
Boxplots of risk scores. Legend: 0 = Omega-3 index < 3% 1 = Omega-3 index > 3% Fig. 2**a**: Dry season scores for omega-3 index. Figure 2**b**: Wet season scores for omega-3 index. Figure 2**c**: Average score across seasons for omega-3 index

Using the logistic regression coefficients, we calculated scores for those ‘at risk’ of an omega-3 index < 3% and those ‘not at risk’, by season (see Supplementary Material). By averaging across the seasonal scores, we also calculated scores for those ‘never at risk’, ‘seasonally at risk’ (omega-3 index < 3% in one season only) and ‘chronically at risk’ (omega-3 index < 3% in both seasons). There appeared to be a clear trend in scores differentiating between those ‘chronically at risk’ and those ‘never at risk’, thus demonstrating the value of the model to identify the most vulnerable. Both groups included girls from each religion (Muslim and Hindu) and across all salinity areas, but proportionately more Hindu women were ‘never’ at risk and less prone to ‘seasonal’ and ‘chronic’ risk. Adolescent girls living in medium salinity areas were least ‘at risk’ overall. Adolescent girls in low salinity and freshwater areas had the highest risk of being ‘chronically at risk’. Girls ‘chronically at risk’ had a diet diversity score and tilapia intake at lower levels than those never at risk, with differences more pronounced in the dry season. Using anthropometric measures, there was little difference in the proportion of girls between BMI categories ‘at risk’ i.e. with an omega-3 index < 3%. Although there is some overlap in scores since the metric cannot predict risk with full accuracy, a cut-off score could be set to identify those ‘at risk’, i.e. a value that dichotomises the result of the metric to a simple binary decision by treating the values above and below a specific threshold as ‘at risk’ or not. For example, averaging across the year, a cut-off of around 2.0 of the composite score calculated from the answers to a range of different questions would identify most girls ‘chronically at risk’ and few ‘never at risk’ (Fig. 2).

## Discussion

The main aim of this research was to establish links between agro-ecological and socio-economic factors, fish intake and nutritional wellbeing in adolescent girls in Bangladesh. The omega-3 index has been proposed as a biomarker for cardiovascular (CVD) risk [28] and as a biomarker of fish intake—but then mainly of oily fish intake [36]. It is not expected that changes in the omega-3 index are associated with anthropometrics and other micronutrient biomarkers and, therefore, it should not be put forward as a general marker of nutritional deficiency. However, for this specific population, where fish intake is relatively high and important, the omega-3 index could present a different but also sensitive marker of nutritional status in relation to fish intake (where micronutrients may be slightly less sensitive).

Evidence from previous studies indicates that the omega-3 index has clinical relevance, being not only a biomarker of fish intake but now also emerging as a risk factor for fatal and non-fatal cardiovascular events in high-income countries [28] and depression and pre-term birth in a wide range of countries, including LMIC [37]. The observational data enabled us to develop and validate a metric, a user-friendly tool that offers a way of predicting omega-3 status by deriving an immediate risk score from responses to a few short questions on religion, salinity zone, female autonomy, diet diversity and tilapia consumption. As stated above, a composite score is calculated from the response of each girl to these questions multiplied by the logistic regression coefficients for the relevant season in which the questions are asked. This algorithm can be set up online to enable fieldworkers to simply enter the response data. The use of a metric using a few short questions is cheaper, can be done online, and avoids the complexity and cost of finger prick blood sampling and biomarker measurement based on field samples. It is ideal in contexts where the relationship between these variables has already been established such as large areas of coastal Bangladesh but would need to be reworked based on the same process for different sociological and geographical contexts.

Also, the study provides a more detailed insight into the factors of importance in health and wellbeing of adolescent girls, including modifiable risk factors, and thus ways to potentially intervene. For example, low omega-3 index levels could be an indication of general low levels of fish being accessible in the food environment or, of a lack of female autonomy, which is, in itself, challenging to measure as surveys may have to be redesigned to each specific setting and/or be controversial in some settings.

We found that the metric discriminates better in the wet season compared with the dry season, reflecting that during the dry (or lean) season, nutritional deficiency is more widespread. During this season, any interventions to address nutritional deficiencies would more likely be at a population level, rather than identifying individuals at risk. Given the seasonal impact on food intake [10, 11], this is a significant finding as the metric would enable interventions to be targeted during the wet season to those girls who are most likely to remain chronically ‘at risk’ of nutritional deficiency. The influence of seasonality on food consumption is widely acknowledged [38]. In rural Timor Leste, fish consumption was higher in coastal than inland locations during the wet rather than dry season [39]. Karim and Little [40] found household consumption and income from freshwater fish culture was strongly impacted by season and household wellbeing status in Northern Bangladesh. In relation to seafood consumption specifically, Karageorgou et al. [4] found differences in intake by religion (Muslims versus Christians, Hindus) which was not observed for other food groups [4]. However, whilst differences in seasonality and religion exist, there is still much unexplained variability in the model. Other factors such as genetics or other phenotypic variables that were not measured may also play a role.

Similar to previous research [10, 11], we found that anthropometry outcomes did not show consistent correlations with the omega-3 index, highlighting the challenges of measuring nutritional wellbeing in adolescents [41]. It is generally accepted that a limitation of anthropometric measurements is that they are unable to identify protein and micronutrient deficiencies, detect small disturbances in nutritional status, nor identify small changes in the proportions of body fat to lean body mass [42, 43]. There have been calls for the use of alternative methods to measure dietary quality among adolescents, complementing anthropometry with the systematic collection of other information, particularly dietary intake [44]. We found that the intake of the main fish fatty acids eicosapentaenoic acid (EPA) and docosahexaenoic acid (DHA), correctly reclassifies adolescent girls from intermediate to high or low risk in our specific setting in Bangladesh which is an important criterion for a novel biomarker [45].

This research is important for aquaculture-ecozones such as coastal Bangladesh. The use of different aquaculture-ecozones defined by salinity in our study setting has enabled us to establish relationships between seasonal fish intake, the role of women’s autonomy and health outcomes. This study has improved our understanding of factors including salinity, seasonality and within-household/community social contexts and health outcomes. We have established that higher female (ie adolescent girls’) autonomy, religion (being Hindu rather than Muslim), geographical location (i.e. living in a high or mid-saline area), and a higher dietary diversity were the strongest predictors of whole blood omega-3 index, an established marker of fish intake but also a risk marker of cardiovascular disease [46], in adolescent girls in the Khulna area of Bangladesh. Whilst being a major contributor to the intake of dietary energy, fat and protein, fish consumption also lowers the risk of coronary heart disease and stroke [47, 48], which may become increasingly relevant for the treatment and prevention of non-communicable diseases in LMICs. Fish production and availability in agro-ecological coastal zones is dependent on seasonal and annual fluctuations in freshwater supply creating a variable salinity gradient, which impacts on aquatic food production, and on food production more generally. The local communities living in these dynamic aquatic eco-zones are vulnerable to poverty, poor diet and health, and whilst these ecosystems produce highly valuable and nutritious aquatic foods, changes to the supply and accessibility of aquatic foods may impact on the dietary quality of the population, as well as on population health outcomes [1].

The main purpose of developing a metric, a statistical model used to predict the likelihood that an individual with a given set of risk factors will experience a health outcome [49], is to provide an effective tool to reveal these specific linkages in practice. Its application would enable the development and implementation of better informed and more integrated policies and practices in relation to aquatic food production systems and inform the design of interventions that might be appropriately targeted to promote healthy fish-containing diets and aquatic agro-ecosystems. It is anticipated that these findings will be potentially relevant for other coastal LMIC that are dependent on fish, particularly freshwater fish, to increase dietary diversity and ensure sufficient intake of energy, macro- and micronutrients. As such, the metric is intended for use in local communities living in such dynamic aquatic eco-zones or geographic areas with similar coastal saline deltas with a high concentration of people and extensive aquaculture.

The importance of social and cultural norms on intra-household access to nutrients and micronutrients in South Asia generally has long been understood [50]. Including the omega-3 index and women’s autonomy score, a key indicator of resource allocation within the household [8, 51–53], in this metric are novel additions to complement existing indicators such as anthropometry. Adolescent girls from Hindu communities enjoyed more autonomy in terms of mobility, ambition and food choice compared to individuals from Muslim communities. The inclusion in the metric of one type of fish, tilapia, that are strongly correlated with omega-3 index demands further analysis. Tilapia was introduced in Bangladesh but is now occurring as natural breeding populations in ponds. Tilapia freely breeds in saline ponds for frequent harvest supporting local consumption whereas it is excluded from freshwater ponds where it is believed to compete with the key economic species, *Macrobrachium*. In other regions of Bangladesh, tilapia have been promoted in fresh water and the species has risen to the top three most produced and consumed fish [54]. It is also among the most affordable of farmed fish and consumed by the poorest consumers. In Barisal, Bangladesh, consumption is particularly high in the wet season reflecting greater availability and, if not self-sourced, their affordability [55].

In a wider global context, this research is important for documenting the wellbeing of adolescent girls. The promotion of fish consumption as an important dietary source of protein, micronutrients and vitamins could play a key role in nutritional security in this vulnerable group [56]. Here, we developed a risk-based prediction algorithm to identify adolescent girls at increased risk of nutritional deficiency. By combining readily measured risk factors, as informed by our previous studies [10, 11], we could produce an overall risk score which can inform more targeted and appropriate dietary and health interventions. Currently, there is no metric available that takes account of environmental, cultural and economic contexts when considering dietary health from a health policy perspective. Indeed, policies addressing the specific challenges of nutritional needs of these communities are limited by the sectoral separation of aquatic food production, the broader food sector and public health institutions [1, 2]. There is an urgent need for metrics to incorporate all these aspects. Bennett et al. [44] called for new metrics to better understand current and potential nutrient production, and highlight specifically the need to understand "pathways linking fish to food and nutrition security’’ (direct consumption, income, women's empowerment).

The strength of the study is the use of primary data from a field study designed to develop the specific metric. Also, the validation of the metric by external data collected with the specific purpose is a major strength of the validity of the metric.

The study is limited by a number of assumptions for the predictors. For women’s autonomy, we assumed an equal value for each dimension in our multidimensional measure. This is the most common approach for setting weights. This approach has been used in many examples, including the United Nation’s Human Development Index but, whilst convenient, this approach has been criticised [57] on the basis of assuming that all dimensions are equally valuable to people. For example, in the women’s autonomy score, responses linked to food choice and nutritional food access were as important as opportunities for recreational activities and freedoms in being able to make personal decisions affecting daily life activities. Other data-driven methods to adjust the weights could be employed in future iterations [58].

Micronutrients are not part of the metric as either a predictor or outcome. A manuscript is currently under review which assesses the micronutrient status of the same adolescent girls [59]. In that manuscript, we investigate the status and risk of deficiencies for iron, iodine, vitamin D and vitamin A separately. For the analysis in this study, the status of deficiencies of the four micronutrients assessed are aggregated into a score, rather than including each separately. In this way, we capture a score for generalised micronutrient riskwhich is clearly a cruder analysis. Using a combined count (score) would hide how any deficiencies may potentially differ by salinity site and season for each micronutrient. Similarly, socio-economic status could have been split into better off/worse off categories rather than just splitting by quintiles.

Yet, despite these limitations, the development of this metric, with the participation of potential users in Bangladesh, would have value to development agencies assessing strategies for other coastal or saline affected areas in terms of maintaining agricultural yield and the quality of food systems and nutritional outcomes for vulnerable groups. Its development is an iterative process as we continue to interrogate the data. These data were collected in the relatively small agro-ecological area of Khulna in Bangladesh. In order to enhance the external validity and generalisability of our findings, it is imperative that these data are validated in a larger variety of different agro-ecosystems. Bangladesh has an estimated 30 million people living in agro-ecosystems, including coastal wetlands that are vulnerable to climate change and salinisation, but such transformations are also occurring in other densely populated LMICs in Asia and further afield where we intend to validate and refine the metric further. There is also the potential for linking this metric to a future economic or policy model with costs and resource use included to evaluate the cost-effectiveness of interventions.

## Conclusion

In adolescent girls in the Khulna area of Bangladesh, we have found that geographical region, salinity, seasonality and higher female autonomy are predictors for impacts of farmed seafood-producing agro-ecosystems on whole blood omega-3 index, a risk marker of cardiovascular disease and associated health outcomes. These adolescent girls represent a highly vulnerable group due to the double nutritional requirements: for their own development, as well as their infants. Improving female decision-making autonomy could positively impact on their dietary intake, indicating an important role for female autonomy in food access and food choice, nutritional status and health. We have developed a metric that contributes to our understanding of factors affecting the nutritional wellbeing of adolescent girls in coastal Bangladesh. As the main aim of a prediction model is to predict risk based on associations between predictors and the outcome, associations should not be interpreted causally. The metric has potential to improve the efficiency of targeting nutritional and health interventions to adolescent girls most at risk from low omega-3 levels in seafood-producing communities. Application of the metric could enable the development and implementation of better informed and more integrated policies and practices in relation to aquatic food production systems, balancing the priorities of enhanced global economic markets versus local food security. This metric will be a valuable tool to be used in a preventative and strategic manner by development agencies and local service providers. The identification of particularly at-risk individuals would improve targeting of timely and cost-effective interventions.

Further research is needed to assess the ability of the metric to predict omega-3 status in a wider setting by validating it in other LMIC whose economic performance is also strongly dependent on its aquatic agro-ecosystem. It is also necessary to improve the assessment of human nutrient status based on fish consumption, with further evaluation of the metric to determine whether it improves outcomes if used in practice.

## Supplementary Information

**Additional file 1.** Supplementary materials S1 doc and S2 doc.

## Data Availability

The datasets generated and/or analysed during the current study are not publicly available due to the data being a key part of a forthcoming PhD but are available from the corresponding author on reasonable request.

## Footnotes

1 See Box 3.7.

2 from -2SD under-and + 2SD overweight.

3 Note this is a population cut off value.

4 A manuscript is currently under review, reporting the investigation of the status and risk of deficiencies for iron, iodine, vitamin D and vitamin A separately.
