# Supplementary material for: Adolescent girls in aquaculture ecozones at risk of nutrient deficiency in Bangladesh development and validation of an integrated metric
Source: BMC Public Health. 2023 Feb 28;23:405. doi: 10.1186/s12889-023-15175-z (PMC9972605; doi:10.1186/s12889-023-15175-z)
Supplement: Supplementary file 1 — Additional file 1. Supplementary materials S1 doc and S2 doc. [file 12889_2023_15175_MOESM1_ESM.docx]

**Supplementary materials**

**S1. Main survey**

Attached questionnaire

**S2. Metric questionnaire and algorithm** **for risk of low omega-3 index in adolescent girls**

**Area**  HS, MS, LS, FW, PP

**Religion** Muslim/Hindu

**Diet diversity**  YES/NO to each of 10 food groups in last 24 hours

**Female autonomy** Do you have any idea about nutrition?

Do you have the chance to gain nutritional knowledge?

Do you think you get enough opportunities and time for recreational activities? (choice of multiple options)

**Fish intake** On how many days did you or your family consume tilapia in last week

$\boldsymbol{Risk algorithm low omega Dry Season}$

*(Wet Season using corresponding co-efficients in Table 2)*

*HS = 0.169 + 0.752* (if Hindu) + 0.256*diet diversity score - 0.103* Female autonomy (mobility) - 0.077*Female autonomy (knowledge) - 0.021*Tilapia count*

*MS = 0.169 + 0.752*(if Hindu) + 0.256*diet diversity score - 0.103* Female autonomy (mobility) - 0.077*Female autonomy (knowledge) - 0.021*Tilapia count + 2.035*(if MS)*

*LS = 0.169 + 0.752*(if Hindu) + 0.256*diet diversity score - 0.103* Female autonomy score (mobility) - 0.077*Female autonomy score (knowledge) - 0.021*Tilapia count + 0.220*(if LS)*

*FW = 0.169 + 0.752*(if Hindu) + 0.256*diet diversity score - 0.103* Female autonomy (mobility) - 0.077*Female autonomy (knowledge) - 0.021*Tilapia count - 0.885*(if FW)*

*PP = 0.169 + 0.752*(if Hindu) + 0.256*diet diversity score - 0.103* Female autonomy (mobility) - 0.077*Female autonomy (knowledge) - 0.021*Tilapia count -0.409*(if PP)*

**S3. Blood / urine analysis**

Blood and spot urine samples (for urinary iodine) were collected by trained medical technologists from International Centre for Diarrhoeal Disease and Research (icddr,b). In both survey points, blood and urine samples were collected in a health centre, developed as temporary sample collection centres on the same day. To obtain 1200 µl serum, 3.5 ml of venous blood was collected in a Venoject tube. After the blood collection from all participants, the blood tubes were placed in a cool box and allowed to clot. At the end of each day, the whole blood was centrifuged and the serum aliquoted into at least three cryovials by pipetting using a disposable pipette. Barcoded label was provided for each of the study participant’s questionnaire forms and each of the aliquoted cryovials. The serum was stored in a freezer (-20ºC or colder) as soon as possible. During the transportation of the serum to the Nutritional Biochemistry Laboratory at icddr,b, the cold chain was maintained. Samples were stored in a -70⁰C freezer and analyzed in the Nutritional Biochemistry Laboratory to estimate blood parameters. The participants provided urine samples in single-use plastic cups from the survey spot. The samples were transferred to wide-mouthed screw-capped plastic bottles that had been previously washed with de-ionized water and dried.

Whole blood was collected using the finger prick method (Accu-Chek® Safe-T-Pro Plus auto lancet; Roche Diagnostics GmbH. Mannheim, Germany), with the first drop of blood wiped and the subsequent blood drops collected on PerkinElmer 226 spot saver cards (PerkinElmer Health Sciences Inc., Greenville, South Carolina, USA), pre-treated with BHT (50 mg/100 ml in ethanol). Blood spots were air dried before transferred to zip-lock foil bags with silica sachets. Samples were stored at -20^o^C for a maximum of two weeks prior to shipping to the University of Stirling where they were analysed within 24 hr upon arrival. Sample spots were removed from the collection card and placed into screw-cap vials before undergoing direct methylation using 1.2M HCl in methanol for 1 h at 70^o^C, aided by a bespoke automated PAL HTX-xt robotic arm (CTC Analytics AG, Zwingen, Switzerland), based on the methods of others (Bell et al., 2011; Marangoni et al., 2004). Fatty Acid Methyl Esters (FAME) were extracted with isohexane and purified by adsorption chromatography using 6 ml 500 mg sorbent acid washed silica solid-phase extraction cartridges (Clean-up® silica extraction columns; UCT, Bristol, Pennsylvania, USA). FAME were separated and quantified by gas liquid chromatography (GLC) using a Thermo Finnigan Trace GC (Thermo Scientific, Milan, Italy) equipped with a 60 m × 0.32 mm × 0.25 mm ZB-wax capillary column (Phenomenex, Cheshire, UK) Helium was used as carrier gas at constant flow (0.8 ml/min) with injector temperature at 250^o^C. The GLC temperature programme was from 50 to 150^o^C at 4^o^C.min^-1^, then to 210^o^C at 1.5^o^C.min^-1^, 271^o^C at 0.5^o^C.min^-1^ to a final temperature of 240^o^C at 40^o^C. Individual FAME were identified compared to in-house standards as well as commercial FAME mixtures (Restek 20-FAME Marine Oil Standard; Thames Restek Ltd., Buckinghamshire, UK).

**S4. Output exploring correlations between ‘nutritional outcomes’ ie omega-3 index, anthropometrics and other micronutrient biomarkers – and responsiveness to changes in salinity (wet season).**

Equation Obs Parms RMSE "R-sq" F P>F

MUAC 256 5 23.06504 0.0507 3.353934 0.0107

BMI_for_Age 256 5 1.133287 0.0190 1.213815 0.3054

BMI 256 5 2.901375 0.0319 2.069352 0.0853

Vit_D^^[[1]](#footnote-1)^^ 256 5 15.22689 **0.2563** 21.62335 **0.0000**

Urin_iodine^^[[2]](#footnote-2)^^ 256 5 465.8011 **0.2912** 25.77397 **0.0000**

Ferritin^^[[3]](#footnote-3)^^ 256 5 35.3647 0.0134 .8523759 0.4932

MN_score^^[[4]](#footnote-4)^^ 256 5 .4911763 0.0435 2.853629 0.0243

omg_3_index 256 5 .808666 **0.3438** 32.87976 **0.0000**

Correlation matrix

MUAC BMI_for_Age BMI Vit_D Urin_iodine Ferritin_R1 MN_scor omg_3_index

MUAC 1.0000

BMI_for_Age 0.7864* 1.0000

BMI 0.8530* 0.9273* 1.0000

Vit_D^1^  0.0173 -0.0383 0.0125 1.0000

Urin_iodine^2^ 0.0336 0.0491 0.0733 -0.0632 1.0000

Ferritin^3^ 0.1017 0.0172 0.0434 0.1344 0.1220 1.0000

MN_score^4^ 0.0850 -0.0030 0.0345 -0.0042 0.1310 0.4539** 1.0000

omg_3_index 0.1023 0.1246 0.1674 0.2917** -0.0839 0.0907 -0.0234 1.0000

*high positive, **low positive, <.30 n/a (Hinkle DE, Wiersma W, Jurs SG (2003). Applied Statistics for the Behavioral Sciences 5th ed. Boston: Houghton Mifflin *in* Mukaka MM. Statistics corner: A guide to appropriate use of correlation coefficient in medical research. Malawi Med J. 2012 Sep;24(3):69-71. PMID: 23638278; PMCID: PMC3576830).

Stata code: *mvreg MUAC_R1 BMI_for_Age_R1 BMI_R1 Vit_D_R1 Urin_iodine_R1 Ferritin_R1 MN_scor_R1 omg_3_index_R1 = i.area_R1, corr*

**S5. Output exploring associations between changes in salinity and anthropometrics (both seasons – dry, wet): Sensitivity analysis**

***BMI classified from ‘obese’ (>30 BMI kg/m^2^) to ‘grade III thinness’ (<16 BMI kg/m^2^)**

***Z-scores for age adjusted**

***BMI standard global cut-offs**

***BMI Asian population cut-offs**

***MUAC with cut-off 210mm**

**MUAC with cut-off 185mm**

**S6. Output exploring associations between changes in omega_3 index and anthropometrics (both seasons – dry, wet): Sensitivity analysis**

***BMI classified from ‘obese’ (>30 BMI kg/m^2^) to ‘grade III thinness’ (<16 BMI kg/m^2^)**

***Z-scores for age adjusted**

***BMI standard global cut-offs**

***BMI Asian population cut-offs**

***MUAC with cut-off 210mm**

***MUAC with cut-off 185mm**

**Hosmer-Lemeshow test (dry season)**
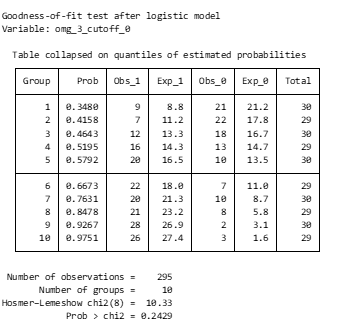


**Hosmer-Lemeshow test (wet season)**


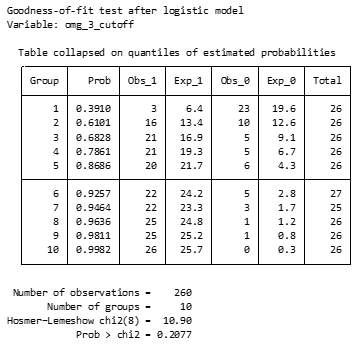


1. Total vitamin (OH)D level was measured by electrochemiluminescence binding assay using a Roche Cobas e601 automated immune analyzer (ref *Ong L, Saw S, Sahabdeen NB et al. (2012) Current 25-hydroxyvitamin D assays: do they pass the test? Clinica chimica acta 413, 1127-1134).* The assay employs a polyclonal antibody directed against 25-OH vitamin D (ref *Leino A, Turpeinen U, Koskinen P (2008) Automated measurement of 25-OH vitamin D3 on the Roche Modular E170 analyzer. Clinical chemistry 54, 2059-2062).* [↑](#footnote-ref-1)
2. Urinary iodine was determined by a colorimetric method at the icddr,b. (ref *Dunn JT, Crutchfield HE, Gutekunst R et al. (1993) Two simple methods for measuring iodine in urine. Thyroid 3, 119-123)*. [↑](#footnote-ref-2)
3. Serum ferritin, C-reactive protein (CRP) and alpha-1-acid glycoprotein (AGP) were analyzed by a sandwich ELISA technique. (ref *Erhardt JG, Estes JE, Pfeiffer CM et al. (2004) Combined measurement of ferritin, soluble transferrin receptor, retinol binding protein, and C-reactive protein by an inexpensive, sensitive, and simple sandwich enzyme-linked immunosorbent assay technique. The Journal of nutrition 134, 3127-3132)*. [↑](#footnote-ref-3)
4. MN score cut-offs: Urinary iodine deficiency (UID) was defined as a excretion level (< 99 µgm/L). (ref *WHO (2013) Urinary iodine concentrations for determining iodine status in populations. World Health Organisation)*. The threshold for vitamin A deficiency (VAD) was a serum retinol concentration of <0.7 µmol/L and severe VAD at <0.35 µmol/L. Vitamin (OH)D deficiency at the population level was defined as a median serum level <50 nmol/L (ref *Del Valle HB, Yaktine AL, Taylor CL et al. (2011) Dietary reference intakes for calcium and vitamin D*). Iron deficiency (ID) at the population level was defined as a median serum level <15 mmol/L, after adjusting for inflammatory markers CRP and AGP (ref: *Ayoya MA, Spiekermann-Brouwer GM, Stoltzfus RJ et al. (2010) α₁-Acid glycoprotein, hepcidin, C-reactive protein, and serum ferritin are correlated in anemic schoolchildren with Schistosoma haematobium. American journal of clinical nutrition)*. [↑](#footnote-ref-4)
